# Correction: Lee et al. Tumor-Associated Macrophages Affect the Tumor Microenvironment and Radioresistance via the Upregulation of CXCL6/CXCR2 in Hepatocellular Carcinoma. *Biomedicines* 2023, *11*, 2081

**DOI:** 10.3390/biomedicines13092271

**Published:** 2025-09-16

**Authors:** Hsin-Lun Lee, Yi-Chieh Tsai, Narpati Wesa Pikatan, Chi-Tai Yeh, Vijesh Kumar Yadav, Ming-Yao Chen, Jo-Ting Tsai

**Affiliations:** 1 Department of Radiology, School of Medicine, College of Medicine, Taipei Medical University, Taipei 11031, Taiwan; 132018@h.tmu.edu.tw; 2 Department of Radiation Oncology, Taipei Medical University Hospital, Taipei 11031, Taiwan; 3 The PhD Program for Translational Medicine, College of Medical Science and Technology, Taipei Medical University and Academia Sinica, Taipei 11031, Taiwan; 4 Department of Radiation Oncology, Cancer Center, Taipei Medical University—Shuang Ho Hospital, New Taipei City 23561, Taiwan; 11445@s.tmu.edu.tw; 5 Division of Urology, Department of Surgery, Faculty of Medicine, Universitas Gadjah Mada, Yogyakarta 55281, Indonesia; narpatiwp@gmail.com; 6 Department of Medical Research and Education, Taipei Medical University—Shuang Ho Hospital, New Taipei City 23561, Taiwan; ctyeh@s.tmu.edu.tw; 7 Continuing Education Program of Food Biotechnology Applications, College of Science and Engineering, National Taitung University, Taitung 95092, Taiwan; 8 Division of Gastroenterology and Hepatology, Department of Internal Medicine, School of Medicine, College of Medicine, Taipei Medical University, Taipei 11031, Taiwan; vijeshp2@gmail.com; 9 Division of Gastroenterology and Hepatology, Department of Internal Medicine, Taipei Medical University—Shuang Ho Hospital, New Taipei City 23561, Taiwan

In the original publication [1], there was a mistake in the Institutional Review Board Statement section of this article. This research project received ethical approval from the Joint Institutional Review Board (JIRB) of Taipei Medical University–Shuang Ho Hospital (Approval No.: JIRB N202203097, date of approval 24 March 2022). The original plan was to collect samples from 50 liver cancer patients, obtained through an application to the National Biobank Consortium of Taiwan (NBCT) and the Taipei Medical University Joint Biobank, including specimens and paraffin-embedded tissue sections (FFPE). However, due to insufficient samples in the biobank, the researchers formally applied to the Department of Pathology at Taipei Medical University-Shuang Ho Hospital, to obtain FFPE samples from 50 deceased liver cancer patients for the experiment.

Unfortunately, the research team did not submit a protocol amendment to the IRB prior to this change at that time, resulting in a non-compliance incident. The researchers have proactively reported the non-compliance (NC) to the IRB and provided a detailed explanation that the collected samples were from 50 deceased patients and were fully de-identified, posing no risk to the individuals. Following the review of the IRB, the researchers were instructed to issue an amendment to the journal editor clarifying that the samples used in the study came from Shuang Ho Hospital, consistent with what was stated in the published article.

We sincerely request the editor’s assistance in revising the Institutional Review Board Statement section of the article. Please update it to read as follows:

**Institutional Review Board Statement:** The study was conducted in accordance with the Declaration of Helsinki, and received ethical approval from the Joint Institutional Review Board (JIRB) of Taipei Medical University–Shuang Ho Hospital (Approval No.: JIRB N202203097, date of approval 24 March 2022). Fifty archived tissue specimens diagnosed as hepatocellular carcinoma (HCC) were obtained from the Department of Pathology at Taipei Medical University–Shuang Ho Hospital.

The authors state that the scientific conclusions are unaffected. All authors have read and agreed to the published version of the manuscript. This correction was approved by the Academic Editor. The original publication has also been updated.
