# Supplementary material for: Tumor-Associated Macrophages Affect the Tumor Microenvironment and Radioresistance via the Upregulation of CXCL6/CXCR2 in Hepatocellular Carcinoma
Source: Biomedicines. 2023 Jul 24;11(7):2081. doi: 10.3390/biomedicines11072081 (PMC10377183; doi:10.3390/biomedicines11072081)
Supplement: Supplementary file 1 [file biomedicines-11-02081-s001.zip › biomedicines-2439323-supplementary.pdf]

Supplementary Data

Supplementary Table S1. List of antibodies and dilution used.

No.	Target	Dilution	Catalog	kDa
1	GAPDH	1:1000	GAPDH (0411K) Mouse mAb SC-27724	37
2	CXCR2	1:1000	Anti-CXCR2 antibody produced in mouse SAB1406027	40.8
3	CD163	1:1000	Anti-CD163 antibody [EPR19518] (ab182422)	150
4	IFN-γ	1:1000	IFN-γ (3F1E3) Mouse mAb #3159	17
5	p-NF-κB p65	1:1000	Phospho-NF-κB p65 (Ser536) (93H1) Rabbit mAb #3033	65
6	NF-κB p65	1:1000	NF-κB p65 (L8F6) Mouse mAb #6956	65
7	p-p38 MAPK	1:1000	Phospho-p38 MAPK (Thr180/Tyr182) (D3F9) XP® Rabbit mAb #4511	43
8	p38 MAPK	1:1000	p38 MAPK (D13E1) XP® Rabbit mAb #8690	40
9	CXCL6	1:1000	CXCL6 Antibody (PA5-116670)	9
10	γ-H2AXa	1:1000	gamma-H2AX Recombinant Rabbit Monoclonal Antibody (BLR053F)	17
11	RAD50	1:1000	Rad50 Antibody #3427	153
12	p-Chk2	1:1000	Phospho-Chk2 (Thr68) (C13C1) Rabbit mAb #2197	62
13	MLH3	1:500	MLH3 Polyclonal antibody #25298-1-AP Proteintec	160
14	MSH3	1:500	Recombinant Anti-MSH3 antibody [RM405] (ab275928)	127
15	ERCC2	1:500	ERCC2 Polyclonal antibody #10818-1-AP Proteintec	80

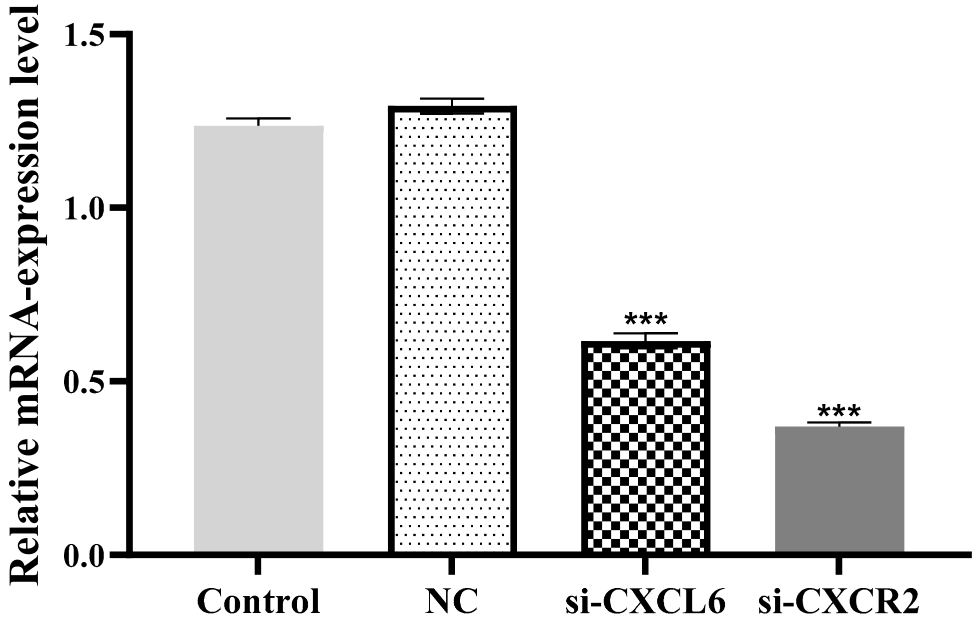

Supplementary Figure S1. Effect of siRNA-CXCL6/CXCR2 on CXCL6 and CXCR2 gene expression. Relative gene expression is the ratio of expression relative to that of untreated control and negative control.

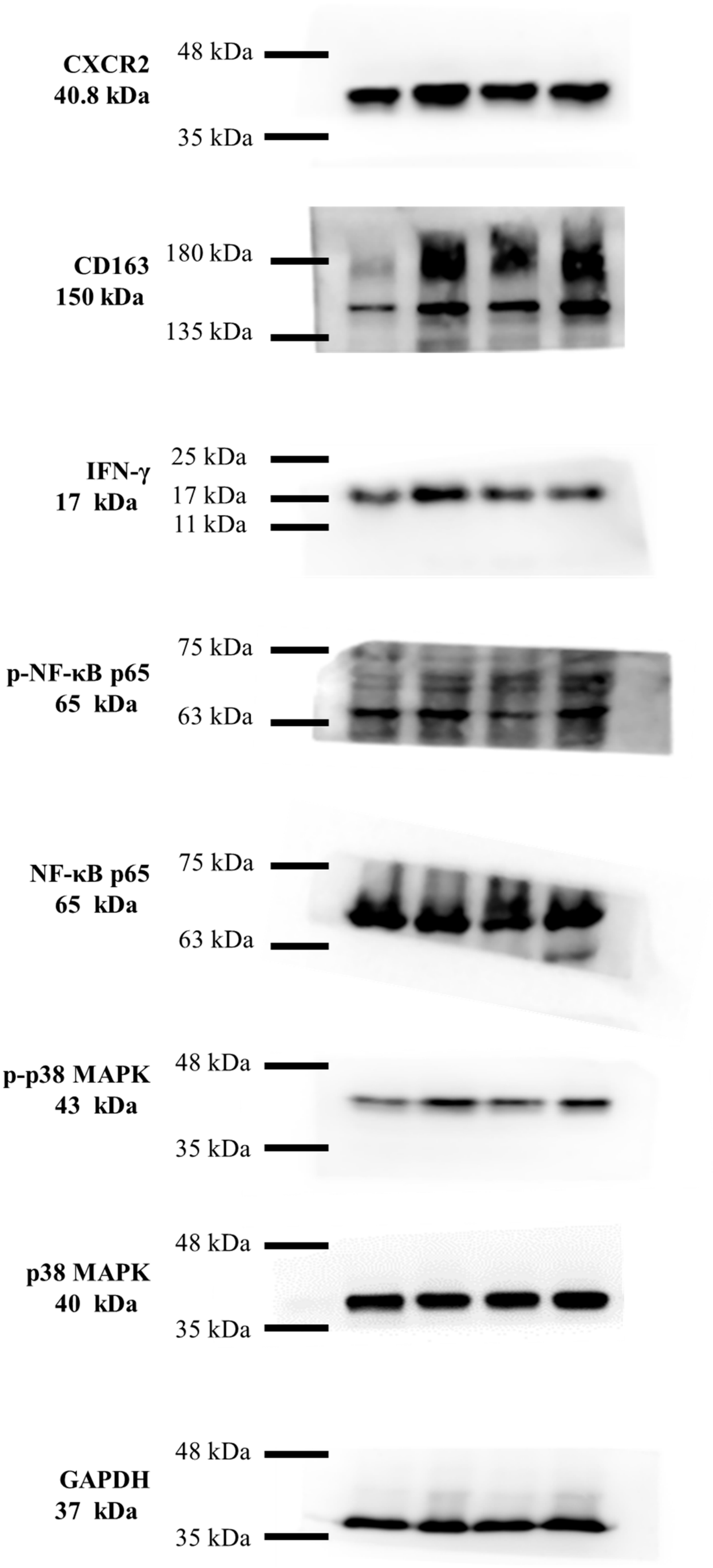

Supplementary Figure S2. Full-size blots of Figure 3D

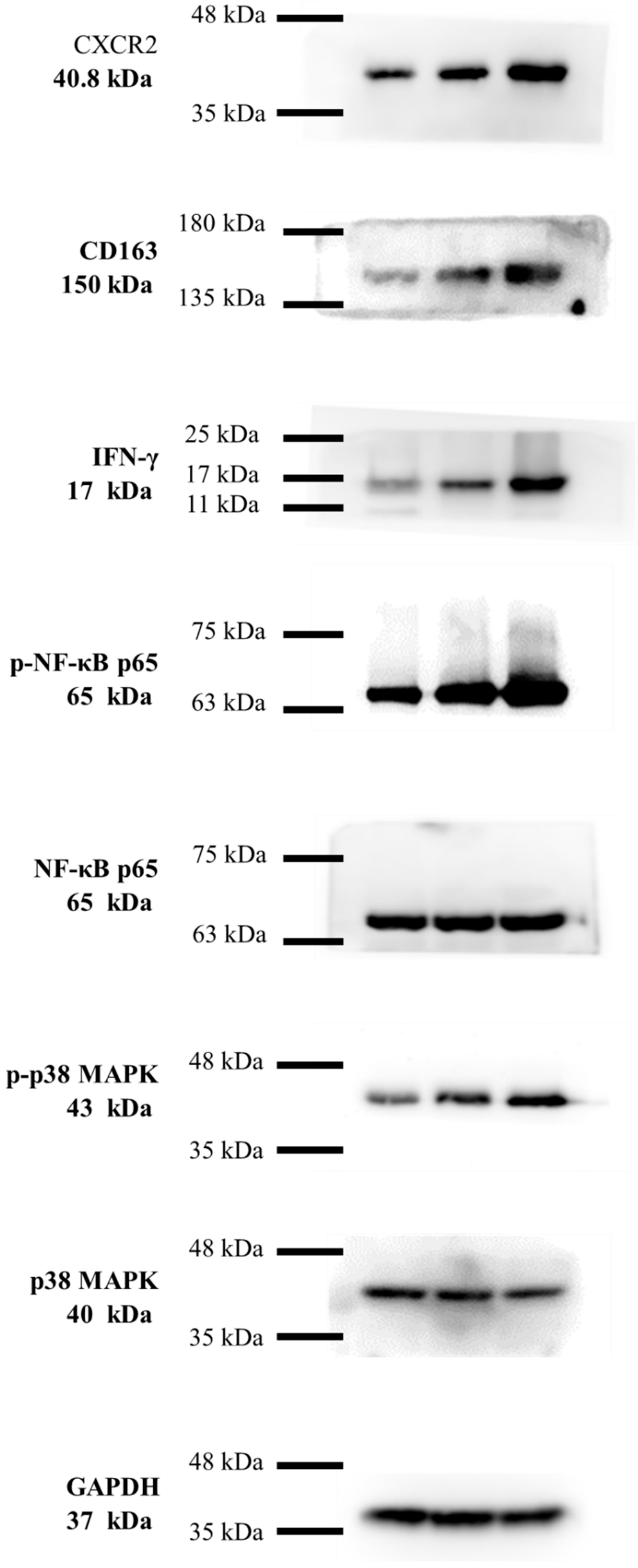

Supplementary Figure S3. Full-size blots of Figure 3E

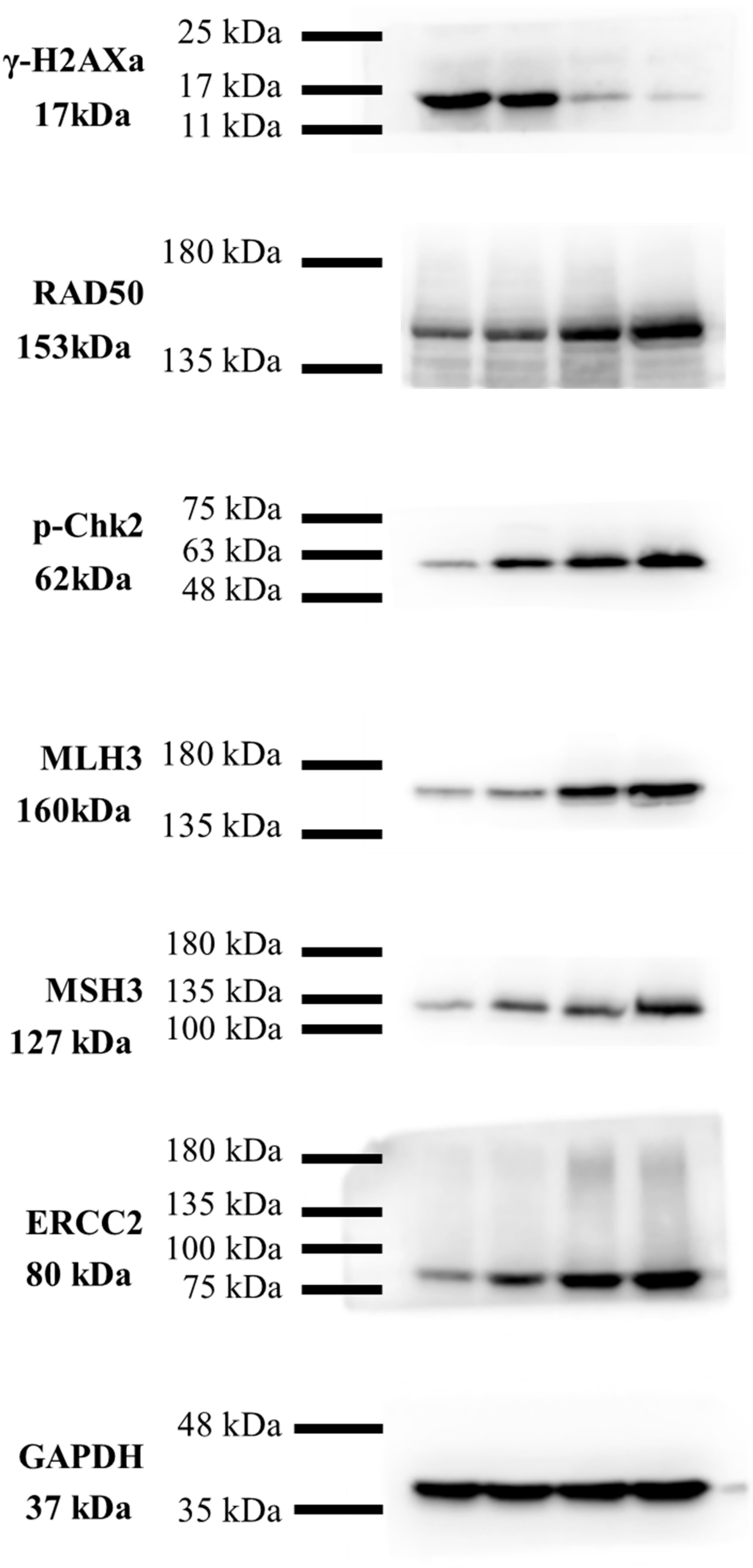

Supplementary Figure S4. Full-size blots of Figure 4B

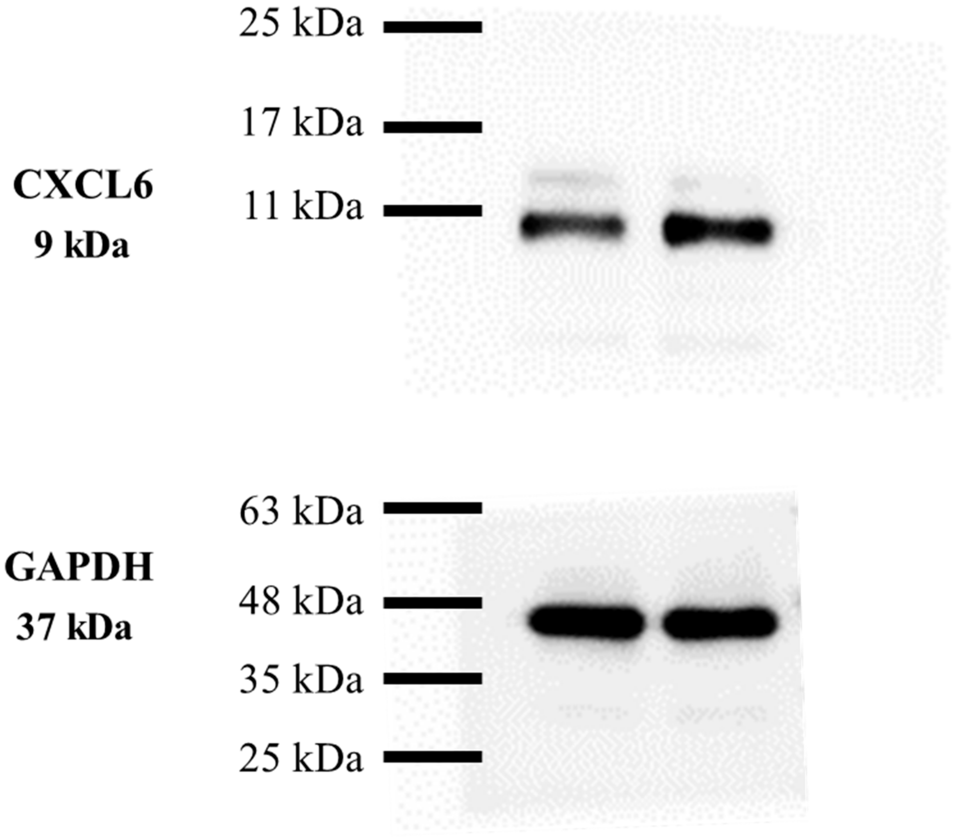

Supplementary Figure S5. Full-size blots of Figure 5A
